# Disentangling the association between alcohol consumption and employment status: causation, selection or confounding?

**DOI:** 10.1093/eurpub/ckac141

**Published:** 2022-10-10

**Authors:** Lluís Mangot-Sala, Nynke Smidt, Aart C Liefbroer

**Affiliations:** Netherlands Interdisciplinary Demographic Institute (NIDI)—Royal Netherlands Academy of Sciences (KNAW), The Hague, The Netherlands; Department of Epidemiology, Faculty of Medical Sciences, University Medical Center Groningen (UMCG), University of Groningen (RUG), Groningen, The Netherlands; Department of Epidemiology, Faculty of Medical Sciences, University Medical Center Groningen (UMCG), University of Groningen (RUG), Groningen, The Netherlands; Netherlands Interdisciplinary Demographic Institute (NIDI)—Royal Netherlands Academy of Sciences (KNAW), The Hague, The Netherlands; Department of Epidemiology, Faculty of Medical Sciences, University Medical Center Groningen (UMCG), University of Groningen (RUG), Groningen, The Netherlands; Department of Sociology, Vrije University of Amsterdam (VU), Amsterdam, The Netherlands

## Abstract

**Background:**

Alcohol use constitutes a major health risk and is related to unemployment. However, the direction of this relationship is unclear: unemployment may change drinking patterns (causation), but heavy drinkers may also be more prone to lose their job (selection). We simultaneously examined selection and causation, and assessed the role of residual confounding. Moreover, we paid attention to the subgroup of abstainers and occupationally disabled, often disregarded in the literature.

**Methods:**

Longitudinal data (three waves collected between 2006 and 2018) of the Lifelines Cohort study from the Netherlands were used (138 875 observations of 55 415 individuals, aged 18–60 at baseline). Alcohol use was categorized as ‘abstaining’, ‘moderate drinking’ and ‘binge drinking’ (≥5 drinks/occasion for male; ≥4 for women). Employment status included occupational disability, short (<6 months) and long-term (≥6 months) unemployment. Random- and fixed-effects multinomial regression models were fitted in order to account for residual confounding. Reciprocal causality was assessed through generalized structural equation modelling with fixed-effects.

**Results:**

Long unemployment spells increase the risk for both binge drinking (*β* = 0.23; 95% CI 0.04–0.42) and abstinence (*β* = 0.27; 95% CI 0.11–0.44), and the effects hold after accounting for reciprocal causality and time-constant confounding. Contrarily, the effect of binge drinking on unemployment is weak (*β* = 0.14; 95% CI −0.03 to 0.31). Abstinence is strongly associated with occupational disability (*β* = 0.40; 95% CI 0.24–0.57).

**Conclusions:**

We find evidence supporting the causation hypothesis (unemployment altering drinking patterns), whereas evidence for the selection hypothesis is weak and mostly confounded by unobserved variables, such as poor health prior to baseline.

## Introduction

Alcohol consumption accounts for 6% of all deaths worldwide^1^ and has been shown to have a complex relationship with health inequalities,^2^^,^^3^ with those with lower socioeconomic status (SES) reporting lower alcohol consumption on average, but experiencing higher rates of alcohol-related harm.^4^ Moreover, acute intoxication is considered a risk factor for cardiovascular diseases, as well as for undesirable social outcomes, such as injuries, violent behaviour, traffic accidents and job loss.^5–7^

There is strong evidence pointing at an association between employment status and alcohol consumption, as the unemployed generally report higher alcohol consumption,^8^^,^^9^ as well as higher rates of harmful drinking patterns, such as binge drinking (BD).^10^^,^^11^ Yet, the direction of the association is still unclear, i.e. whether unemployment increases alcohol consumption or alcohol consumption increases the risk of becoming unemployed.

Several studies have tried to disentangle causality in the association between employment status and alcohol consumption.^10^^,^^12^ Traditionally, two alternative hypotheses emerged: the causation hypothesis, which argues that unemployment may lead to changes in alcohol consumption; and the selection hypothesis, claiming that heavy drinking (HD) may lead to unemployment. In fact, recent evidence tentatively suggests that both processes operate simultaneously,^13^ and that their relative importance is context-dependent^14^ and may vary over the life course.^15^

Moreover, it is often argued in the literature that a significant part of the association is confounded by distal causes—referred to in some studies as ‘indirect selection’^15^^,^^16^—e.g. poor health during early life, which may increase the risk of unemployment and/or disability later in life^17^ and, at the same time, may affect future drinking patterns. Generally, these unmeasured distal causes, which sometimes take place before the observation period, are referred to as ‘residual confounding’.^18^^,^^19^

These issues point to some of the limitations in the literature on the causal association between employment status and alcohol consumption: first, most studies focussed either on causation or on selection^3^^,^^20^^,^^21^ and the few testing both, analysed them as parallel rather than reciprocal processes.^12^ To the best of our knowledge, only one study accounted for reciprocal causality, but it did not account for residual confounding.^10^ Second, most studies did not assess the effect of residual confounding or referred to it when they did not find evidence supporting neither causation nor selection.^14^ Third, studies often compared a majority of ‘moderate’ drinkers with a minority of ‘heavy drinkers’,^10^^,^^22^ glossing over the subgroup of abstainers, i.e. those who do not drink any alcohol, despite the evidence that abstinence is associated with lower SES,^23^^,^^24^ and poorer health.^3^ Fourth, studies often simply juxtaposed the employed and the unemployed, although longer unemployment spells have been shown to have stronger negative effect on health than short unemployment spells.^22^ Finally, most studies did not include ‘occupational disability’ in the analyses, in spite of often being the culmination of a precarious labour-market trajectory.^25^^,^^26^

Our study contributes to the literature in several ways. First, our large longitudinal panel data allows us to test the reciprocal causality in the association, while addressing residual confounding. Second, our analysis of alcohol consumption assesses ‘binge drinking’, a highly risky drinking pattern, strongly patterned by SES^6^ with a great impact on the individual and his/her environment,^1^^,^^7^ as well as the subgroup of abstainers. Third, we incorporate the duration of unemployment spells, as well as individuals who are ‘occupationally disabled’ in the analyses.

We aim at answering the following research questions:

- Does unemployment and/or occupational disability cause changes in alcohol consumption?
- Do changes in alcohol consumption cause changes in employment status and/or disability?

## Methods

### Study design and participants

We used longitudinal data from the Lifelines Cohort, a large population-based prospective cohort study and biobank in the three northern provinces of the Netherlands investigating universal risk factors for multifactorial diseases.^27^ The recruitment of participants was carried out between 2006 and 2013, resulting in a three-generation design (*N* = 167 729). Composition and characteristics of the sample have been discussed elsewhere.^11^^,^^28^ For our study, panel data from waves first (1A); fourth (2A) and fifth (2B) were used (second and third waves did not have complete information on the key variables). Average time between waves was 48.2 months (waves first–fourth); and 21.6 months (waves fourth–fifth). Our study sample included adults aged 18–60 at baseline.

Participants who declared to be full-time students, full-time homemakers, or (early) retired, either at baseline or at follow-up, were excluded, and so were individuals with only one observation or with missing values on the outcome variables, resulting in 138 875 observations of a total of 55 415 individuals (55% female; mean age at baseline 43 years). Sensitivity analyses with imputed data were also performed, showing similar, albeit generally somewhat stronger estimates. However, we opted for the unimputed models in order to facilitate the statistical analyses and enable Generalized Structural Equation Modelling (GSEM). The code used for data imputation is available as a Supplementary file, and the resulting imputed models are shown in Supplementary appendix S1, tables A3 and A4.

### Measurements

#### Outcome variables

*Alcohol consumption*. Two questions referred to frequency and amount of alcohol consumed in the last month: ‘How often did you drink alcoholic drinks in the past month?’ (ranging from ‘not in the last month’ to ‘6-7 days per week’), and ‘On days that you drank alcohol, how many glasses did you drink on average?’ (from ‘1’ to ‘12 or more’). Based on these questions, an average daily alcohol consumption score (glasses/day) was calculated. Those who answered ‘not this month’ to the first question were considered abstainers. Given existing evidence of a non-linear association between unemployment and alcohol consumption,^24^ and recommendations to consider different drinking patterns in the analyses,^7^ alcohol consumption was analyzed as a categorical outcome with three categories: ‘Binge Drinking’, defined as ‘drinking on average ≥5 standard units or more in one occasion (4 for women)’ in the last month,^10^ ‘Moderate (non-binge) drinking’ and ‘Abstention’. Sensitivity analyses with one additional category assessing ‘Heavy drinking’ (‘>1.5 glasses/day’ without BD, following the Dutch Dietary Guidelines^29^) were carried out in order to grasp potential differences between an intensive, intermittent form of drinking (BD) vs. a regular form of mild abuse (HD).

*Employment status*. Unemployment was defined as ‘being unemployed/looking for a job (registered with the employment office)’. ‘Occupational disability’ was based on the Dutch classification system and defined as ‘being disabled for work’, i.e. receiving disability benefits due to long-term illness (thus excluding temporary sick leaves). Employment status was estimated as a categorical variable with four categories: ‘employed’ (in paid employment, full- or part-time), ‘short-term unemployed’ (<6 months), ‘long-term unemployed’ (≥6 months) and ‘occupationally disabled’.

#### Covariates

As time-constant covariates, gender (male/female); and SES, assessed through ‘Years of Education’, using the number of years it would take to complete the respondent’s level of the Dutch educational system by the fastest route possible,^30^ ranging from 5 (>primary school) to 16 years (university education). As time-varying covariates, self-reported health (SRH) status was measured at every wave, ranking from 1 ‘Poor’ to 5 ‘Excellent’; partner status (‘married/partnered’—including living apart together—vs. ‘single/divorced’); and lastly age, in years.

### Statistical analyses

Univariate descriptive analyses of the main variables of interest, as well as bivariate analyses stratified by employment status and alcohol consumption are presented in Supplementary appendix S1, tables A1 and A2.

The analytical strategy for this study consisted of three steps: first, multinomial logistic regression models with robust standard errors clustered by individuals [they will be referred to as random-effect (RE) models] were fitted. These models combine a ‘between approach’ (a comparison between individuals) with a ‘within approach’ (comparing observations of the same individuals over time). Second, fixed-effects (FE) regression models were estimated, which infer the causal effect by examining changes within individuals over time.

Both types of models differ considerably in sample size, as RE models use the whole sample (*n *=* *136 614), whereas FE keep only those individuals whose outcome has changed throughout the observation period (*n* = 38 922). Moreover, in RE models all potential confounders—gender, age, educational level, partner status and SRH—were included, whereas only time-varying confounders—age, partner status and SRH—were added to FE models, as they already account for time-constant confounding. In both cases, alcohol consumption was first analyzed as the outcome variable with employment status as main independent variable (causation). Next, the independent and outcome variable were reversed to test the selection hypothesis. Thus, both directions were analyzed as if they were two independent processes, without accounting for potential reciprocal association.

Hence, in the third stage of our study, cross-lagged models were fitted by means of GSEM techniques, in order to account for the reciprocal association between employment status and alcohol consumption. In these models, the independent variables precede the outcome in time (e.g. unemployment at Wave 1 affects BD at Wave 4, and vice versa). Thus, alcohol consumption and employment status both act as predictor and outcome. Moreover, we estimated these models accounting for FE, based on guidelines of Allison *et al*.^31^ More information about the FE cross-lagged GSEM models can be found in the Supplementary appendix S2.

Apart from the main analyses presented above, we also conducted a number of sensitivity analyses to examine how robust our results were to slight modifications of model specifications. The specific content of these sensitivity analyses is presented in the appropriate section.

To the best of our knowledge, this is the first study to assess the reciprocal association between unemployment and alcohol consumption by means of cross-lagged GSEM models accounting for FE.

## Results

### Causation: unemployment, occupational disability and alcohol consumption

Table 1 shows the estimates for the RE (*n* = 136 614) and FE (*n* = 38 922) models testing the causation hypothesis. While short unemployment spells do not seem to have any effect, long-term unemployment significantly predicts BD (*β* = 0.34; 95% CI 0.26–0.46), as well as abstinence (*β* = 0.36; 95% CI 0.26–0.46). Moreover, these effects hold in the FE model, after accounting for time-constant unobserved heterogeneity (BD: *β* = 0.38; 95% CI 0.13–0.63), although the positive effect of long-term unemployment on abstinence is slightly smaller (*β* = 0.24; 95% CI 0.04–0.44).

**Table 1:** Effects of employment status on alcohol consumption. RE (Model 1) and FE (Model 2)

	Model 1 (RE)		(*n* = 136 614)		Model 2 (FE)		(*n* = 38 922)	
	Abstinence		Binge drinking		Abstinence		Binge drinking	
(ref. moderate drinking)	*β*	95% CI	*β*	95% CI	*β*	95% CI	*β*	95% CI
Employment status (employed)								
Short unemployment	−0.09	−0.21 to 0.03	0.04	−0.10 to 0.18	−0.17	−0.40 to 0.05	−0.02	−0.26 to 0.22
Long unemployment	0.36**	0.26 to 0.46	0.34**	0.21 to 0.46	0.24*	0.04 to 0.44	0.38**	0.13 to 0.63
Occupational disability	0.62**	0.53 to 0.71	−0.02	−0.16 to 0.11	0.57**	0.32 to 0.82	0.02	−0.31 to 0.35
Gender (men)								
Women	0.98**	0.94 to 1.03	−0.72**	−0.76 to −0.67				
Age	−0.02**	−0.02 to −0.02	−0.05**	−0.05 to −0.05	−0.06**	−0.06 to −0.06	−0.05**	−0.05 to −0.04
Health	−0.24**	−0.27 to −0.22	−0.10**	−0.13 to −0.07	−0.07*	−0.12 to −0.02	0.05	−0.01 to 0.11
Partner (partnered/married)								
Single/divorced/widow	0.37**	0.32 to 0.43	0.54**	0.49 to 0.61	−0.16*	−0.30 to −0.02	0.67**	0.53 to 0.81
Years of education	−0.12**	−0.13 to −0.11	−0.17**	−0.18 to −0.16				

* p-value < 0.05; ** p-value < 0.01

In turn, in both models, while occupational disability has no significant effect on BD, it is a significant predictor of abstinence (*β* = 0.62 and *β* = 0.57 in the RE and FE models, respectively). Consistently, self-rated health is negatively associated with abstinence.

In sum, both RE and FE models show that long-term unemployment affects drinking patterns (i.e. it increases both BD and abstinence). Models showed consistently that abstinence is higher among the occupationally disabled. However, the fact that the effect of long-term unemployment on abstinence is substantially smaller in Model 2 suggests that the effect in Model 1 is partially confounded by unobserved confounding.

### Selection: alcohol consumption, unemployment and occupational disability

We now examine the potential causal role of alcohol consumption in increasing the risk of unemployment and/or occupational disability. As shown in table 2, alcohol consumption is not associated with short unemployment spells. However, the RE model (*n* = 136 614) shows that both BD, as well as abstinence, increase the risk of long-term unemployment (*β* = 0.36 both; 95% CI 0.23–0.48 and 0.26–0.46, respectively). In the absence of a feasible explanation why abstinence would increase the risk of unemployment, this could be a spurious effect caused by unobserved variables, e.g. poor health prior to baseline. Consistently, the FE model (*n* = 13 158) shows that, while BD is still a significant risk factor for long-term unemployment (*β* = 0.37; 95% CI 0.11–0.64), the effect of abstinence diminishes drastically (*β* = 0.17; 95% CI −0.04 to 0.39), and becomes not statistically significant.

**Table 2:** Effects of alcohol consumption on short- and long-term unemployment and occupational disability. RE (Model 1) and FE (Model 2)

	Model 1 (RE) (*n* = 136 614)						Model 2 (FE) (*n* = 13 158)					
	Short unemployment		Long unemployment		Occupational disability		Short unemployment		Long unemployment		Occupational disability	
(ref. employed)	*β*	95% CI	*β*	95% CI	*β*	95% CI	*β*	95% CI	*β*	95% CI	*β*	95% CI
Alcohol (moderate)												
Abstinence	−0.09	−0.21 to 0.03	0.36**	0.26 to 0.46	0.54**	0.44 to 0.63	−0.20	−0.43 to 0.02	0.17	−0.04 to 0.39	0.03	−0.28 to 0.35
Binge drinking	0.05	−0.08 to 0.19	0.36**	0.23 to 0.48	−0.01	−0.14 to 0.13	−0.03	−0.27 to 0.21	0.37**	0.11 to 0.64	0.17	−0.24 to 0.59
Gender (men)												
Women	0.05	−0.04 to 0.15	0.07	−0.02 to 0.16	−0.08	−0.17 to 0.02						
Age	−0.01**	−0.02 to −0.01	0.06**	0.05 to 0.06	0.05**	0.05 to 0.06	0.00	−0.01 to 0.01	0.09**	0.08 to 0.10	0.18**	0.16 to 0.20
Health	−0.18**	−0.24 to −0.11	−0.37**	−0.43 to −0.31	−1.59**	−1.65 to −1.52	0.05	−0.06 to 0.16	−0.01	−0.11 to 0.10	−0.45**	−0.61 to −0.28
Partner (partner/married)												
Single/divorced/widow	0.69**	0.57 to 0.80	1.21**	1.12 to 1.31	0.85**	0.74 to 0.95	0.18	−0.08 to 0.44	0.17	−0.10 to 0.44	0.15	−0.32 to 0.62
Years of education	−0.09**	−0.11 to −0.06	−0.12**	−0.14 to −0.10	−0.15**	−0.17 to −0.13						

** p-value < 0.01

Similarly, abstinence appears as a strong predictor for occupational disability in Model 1 (*β* = 0.54; 95% CI 0.44–0.63), but its effect practically disappears in Model 2, after accounting for time-constant unobserved confounding (*β* = 0.03; 95% CI −0.28 to 0.35). BD does not seem to be associated with occupational disability.

It is worth mentioning that sensitivity analyses (see Supplementary appendix S1, tables A5 and A6) show that, when adding an additional category for ‘Heavy Drinking’ into the models, associations with HD are not statistically significant and other results remain practically identical, suggesting that employment status is associated with alcohol intoxication (BD) but not with regular alcohol abuse (HD).

### Reciprocal causality between alcohol consumption and employment status

**Figure 1:**
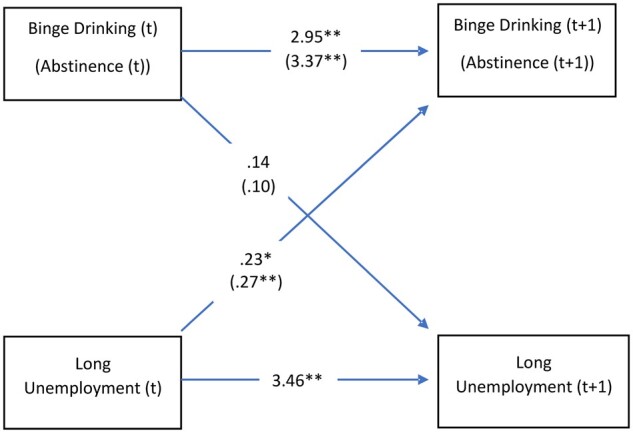
Cross-lagged association between BD, abstinence (between parentheses) and long-term unemployment. Coefficients of FE GSEM model

The third stage of our analyses uses GSEM with FE to simultaneously test both hypotheses about the reciprocal association between alcohol consumption and employment status. As shown in figure 1, after accounting for reciprocal effects, and removing time-constant unobserved heterogeneity in a FE cross-lagged panel model, long-term unemployment still has a significant positive effect on BD (*β* = 0.23; 95% CI 0.04–0.42), as well as on abstinence (*β* = 0.27; 95% CI 0.11–0.44). Sensitivity analyses allowing the coefficients to vary across waves (Supplementary appendix figure A1) show that the effect of unemployment on BD is stronger during the longer period between Waves 1 and 4 (*β* = 0.53; 95% CI 0.27–0.79), whereas the effect of long-term unemployment on abstinence is somewhat stronger between Waves 4 and 5 (*β* = 0.30; 95% CI 0.08–0.52). Lastly, neither BD nor abstinence shows a significant association with long-term unemployment.

**Figure 2:**
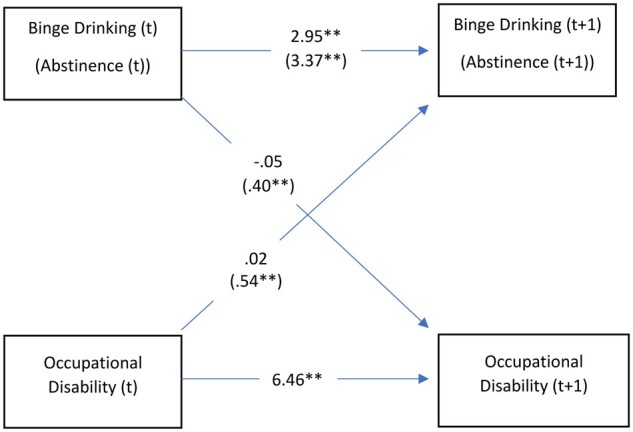
Cross-lagged association between BD, abstinence (between parentheses) and occupational disability. Coefficients of FE GSEM model

The association between occupational disability and drinking patterns shows quite a different picture: BD does not seem to cause or be affected by occupational disability, whereas occupational disability is positively associated with abstinence (*β* = 0.54; 95% CI 0.40–0.67). Moreover, abstinence also appears as a risk factor for occupational disability (*β* = 0.40; 95% CI 0.24–0.47), as shown in figure 2 and Supplementary appendix figure A2.

## Discussion

This is the first study to assess the reciprocal association between employment status and alcohol consumption while accounting for time-constant unobserved confounding. One of its main strengths is that it compares RE and FE models. That enabled us to move from a general picture of the total study population to the inference of causal effects comparing only observations within the same individuals over time.

Our results supported the causation hypothesis, as long unemployment spells increased the risk for both BD and abstinence. However, our results offer limited support for the selection hypothesis, as effects of alcohol consumption on employment status were strongly confounded by unmeasured confounders.

With regard to the effect of long unemployment spells on BD, the evidence is robust, as it held in the FE models, suggesting that BD is a reaction to long unemployment, as previously reported in several studies.^8^^,^^17^^,^^22^ Furthermore, this effect also remained after accounting for reciprocal causality, differing from the results reported by Berg *et al*. (2017).^10^ who, using a similar cross-lagged panel model, found no evidence for unemployment increasing BD. However, these differences may be explained by methodological issues: while their study relied on two birth cohorts and a much younger study population, ours is based on a population-based cohort and an older sample. Moreover, the fact that we removed time-constant confounding, while their results are based on REs GSEM models, suggests that their results may be partly confounded by unobserved variables.

Long unemployment spells also seemed to increase the likelihood of abstinence. Although we find precedents in the literature,^24^^,^^25^ most studies overlooked the subgroup of abstainers, thus missing this potential association with employment status.^10^^,^^22^^,^^32^ Potential explanations range from an income drop^33^ to a reduction in the social network—and social drinking—or an attempt to improve one’s chances on the labour market.^34^ Moreover, occupational disability also increased the odds for abstinence, suggesting a crucial role of poor health in the causal pathway between occupational disability and abstinence.^3^

Our results offered weak support for the selection hypothesis: although initial models showed that BD was a risk factor for long-term unemployment, this effect disappeared when accounting for reciprocal causality. Unlike previous studies that supported the selection hypothesis by means of FE models,^15^ our results suggested that previous evidence supporting this hypothesis^3^^,^^10^ may in fact be confounded by common distal causes—e.g. poor health prior to baseline—that would increase the risk for both BD and unemployment. Alternatively, an alleged effect of BD may also be confounded by lagged effects of previous unemployment spells.

This study relied on a comparison between RE and FE models. One major advantage of this comparison is that it allows to identify residual confounding. However, a downside is that FE only includes individuals whose outcome has changed throughout the observation period. For that reason, both the sample size and composition differed between these models, and the FE models represented a rather deprived subsample—especially when employment status is the outcome—in which those hit by long-term unemployment (2.4% of the sample) and occupational disability (3.7%) were overrepresented. Thus, conclusions regarding the selection hypothesis must be drawn being aware of this fact.

Our comparison between RE and FE models allowed us to identify a spurious effect that would have been otherwise overlooked: abstinence increased the risk for unemployment in the RE models. Given that abstinence is much more common among those with poor health,^3^ this effect may be ‘signalling’ residual confounding: e.g. poor health prior to baseline may increase the risk of abstinence, as well as of unemployment. Consistently, our analyses showed that the positive effect of BD on long-term unemployment became diluted if abstainers were not assessed separately (Supplementary appendix S1, table A8). These findings point at a group of binge drinkers, whose increased risk of unemployment is due to their drinking patterns, as well as a subgroup of abstainers, whose higher risk of unemployment is probably related to pre-existing poor health conditions.

Nevertheless, our results still showed an association between abstinence and occupational disability. There are several possible explanations for that: first, it could be argued that the effect may be caused by lagged effects of unemployment or occupational disability, which increased the odds for both abstinence and occupational disability later on. Moreover, disability can be a long process, in which poor health may lead to numerous sick-leave events before leading to long-term occupational disability. In that context, our variables may not capture the full complexity of the process. In any case, it seems clear that poor health plays a key role in the association between employment status and alcohol use.^17^

Last but not least, our results supported the relevance of distinguishing between short-term and long-term unemployment, as most of the effects were only visible for long-term unemployment. That confirms the idea that the negative effects of unemployment accumulate over time,^8^^,^^17^^,^^22^^,^^35^ and that short-term unemployment may have a weaker negative impact on health (behaviours).^8^

This study has several limitations. First, data did not allow to rely on gold-standard screening tests for alcohol use disorders. However, we relied on widely used indicators, such as BD.^10^^,^^22^^,^^36^ Second, the assessment of abstainers as those who did not drink alcohol in the last month may be sensitive to seasonal variation. Yet, our results showed that it makes sense to distinguish the subgroup of abstainers from those of the moderate drinkers, despite the data limitations. Third, for the purpose of this study, individuals with only one observation were excluded in order to be able to run FE models, and observations with missing values in the outcomes were dropped. That may lead to some attrition bias, since being male, lower educated, single and having poorer health increased the risk of attrition. However, imputed models (Supplementary appendix S1, tables A3 and A4) showed very similar results (with somewhat stronger estimates in some cases), suggesting that the presented effects may, in fact, slightly underestimate the real effects. Fourth, FE models relied on the assumption that educational level was time-constant and it could be argued that educational level may change during the observation period. However, this was the case only for few individuals and we prioritized parsimony in the FE models. Last, our assessment of self-rated health did not allow to distinguish between physical and mental health, nor provided information prior to baseline. Future research should address these issues, with a focus on disentangling the causal role of poor health early in life.

In summary, this study provides robust evidence for the causation hypothesis: long-term unemployment is a risk factor for BD. Moreover, long-term unemployment, and especially occupational disability are associated with higher odds of abstinence. However, evidence supporting the selection hypothesis is weak, and mostly due to unmeasured confounding. A key contribution of this study is that it compares RE, FE and cross-lagged GSEM models in order to identify residual confounding in the reciprocal association between employment status and alcohol consumption.

## Supplementary data

Supplementary data are available at *EURPUB* online.

## Funding

This work was supported by the Research Fund of the Royal Netherlands Academy of Arts and Sciences (KNAW-Institutes).

*Conflicts of interest*: None declared.

Key points

- Results show that unemployment is a risk factor for binge drinking, while it also increases the odds for abstinence in some individuals.
- There is weak evidence for alcohol consumption leading to greater risk of unemployment.
- Unmeasured poor health seems to be confounding the association between employment status and alcohol consumption.
- Public health policies should aim at tackling long-term unemployment as one of the main social determinants of health.

## Supplementary Material

ckac141_Supplementary_Data

## Data Availability

The data underlying this article were provided by Lifelines under licence. Access to the data can be granted under licence by Lifelines and the authors will share their codes used to produce the results presented in this article upon request.
