# Supplementary material for: Disentangling the association between alcohol consumption and employment status: causation, selection or confounding?
Source: Eur J Public Health. 2022 Oct 10;32(6):926–32. doi: 10.1093/eurpub/ckac141 (PMC9713390; doi:10.1093/eurpub/ckac141)
Supplement: ckac141_Supplementary_Data [file ckac141_supplementary_data.zip › ejph-2021-11-om-1082-File005.docx]

**Appendix**

The following appendix contains two parts:

- Appendix #1 includes tables and figures referred to but not included in the main body of the text.

Appendix #2 includes a brief description of the fixed-effects cross-lagged GSEM model.

**Appendix #1 Additional Tables & Figures**

**Table A1. Characteristics of the study population. Univariate descriptive analyses.**

|  | **Baseline** (n=56,249) | | |  | **Wave 4** (n=50,275) | | |  | **Wave 5** (n=37,236) | | |
| --- | --- | --- | --- | --- | --- | --- | --- | --- | --- | --- | --- |
|  | **n (%)** |  | **missing (%)** |  | **n (%)** |  | **missing (%)** |  | **n (%)** |  | **missing (%)** |
| Gender |  |  | 0 (0.00%) |  |  |  | 0 (0.00%) |  |  |  | 0 (0.00%) |
| Male | 25,357 (45.08%) |  |  |  | 22,463 (44.68%) |  |  |  | 16,813 (45.15%) |  |  |
| Female | 30,892 (54.92%) |  |  |  | 27,812 (55.32%) |  |  |  | 20,423 (54.85%) |  |  |
| Age (mean; SD) | 43.09 (8.64) |  | 0 (0.00%) |  | 47.13 (8.56) |  | 0 (0.00%) |  | 49.37 (8.59) |  | 0 (0.00%) |
| Partner Status |  |  | 12 (0.02%) |  |  |  | 0 (0.00%) |  |  |  | 74 (0.20%) |
| Partnered/Married/LAT | 49,020 (87.15%) |  |  |  | 44,386 (88.29%) |  |  |  | 32,719 (87.87%) |  |  |
| Single/Divorced/Widow/Other | 7,217 (12.83%) |  |  |  | 5,889 (11.71%) |  |  |  | 4,443 (11.93%) |  |  |
| Education Years (mean; SD) | 12.45 (2.29) |  | 836 (1.49%) |  | 12.43 (2.29) |  | 745 (1.48%) |  | 12.45 (2.30) |  | 562 (1.51%) |
| Self-reported Health (1=poor; 5=excellent) | 3.42 (0.79) |  | 9 (0.02%) |  | 3.35 (0.81) |  | 77 (0.15%) |  | 3.34 (0.81) |  | 93 (0.25%) |
| Employment Status |  |  | 0 (0.00%) |  |  |  | 0 (0.00%) |  |  |  | 0 (0.00%) |
| Employed | 52,800 (93.87%) |  |  |  | 46,195 (91.88%) |  |  |  | 33,960 (91.20%) |  |  |
| Short Unemployment | 901 (1.60%) |  |  |  | 796 (1.58%) |  |  |  | 431 (1.16%) |  |  |
| Long Unemployment | 1,014 (1.80%) |  |  |  | 1,364 (2.71%) |  |  |  | 1,068 (2.87%) |  |  |
| Occupational Disability | 1,534 (2.73%) |  |  |  | 1,920 (3.82%) |  |  |  | 1,777 (4.77%) |  |  |
| Alcohol Consumption |  |  | 0 (0.00%) |  |  |  | 0 (0.00%) |  |  |  | 0 (0.00%) |
| Abstainer | 11,125 (19.78%) |  |  |  | 7,688 (15.29%) |  |  |  | 6,879 (18.47%) |  |  |
| Moderate Drinking | 38,289 (68.07%) |  |  |  | 37,286 (74.16%) |  |  |  | 26,739 (71.81%) |  |  |
| Binge Drinking | 6,835 (12.15%) |  |  |  | 5,301 (10.54%) |  |  |  | 3,618 (9.72%) |  |  |

**Table A2. Distribution of the population, by alcohol consumption and by employment status. Pooled observations from baseline, wave 4 and wave 5.**

|  | **Abstainer** | **Moderate Drinker** | **Binge Drinker** |  | **Employed** | **Short Unemp**. | **Long Unemp.** | **Disability** |
| --- | --- | --- | --- | --- | --- | --- | --- | --- |
|  | *(n=24,736)* | *(n=99,360)* | *(n=14,779)* |  | *(n=128,328)* | *(n=2,097)* | *(n=3,328)* | *(n=5,122)* |
| Gender (% male) | 24.84% | 47.20% | 64.27% |  | 45.16% | 43.82% | 42.49% | 44.14% |
| Years of Education (mean) | 12.09 | 12.67 | 11.91 |  | 12.56 | 12.12 | 11.65 | 11.30 |
| Partner status (% single) | 15.99% | 10.27% | 18.19% |  | 11.06% | 20.60% | 28.89% | 24.54% |
| Health (% "poor/mediocre") | 14.72% | 7.90% | 9.49% |  | 7.39% | 12.59% | 18.05% | 49.87% |
| Short unemployment (%) | 1.48% | 1.47% | 1.84% |  |  |  |  |  |
| Long unemployment (%) | 3.23% | 2.10% | 2.98% |  |  |  |  |  |
| Occupational Disability (%) | 6.78% | 2.97% | 3.32% |  |  |  |  |  |
| Abstinence |  |  |  |  | 17.06% | 17.50% | 24.04% | 32.76% |
| Binge Drinking |  |  |  |  | 10.58% | 12.97% | 13.22% | 9.59% |

**Table A3. Imputed model. Causation hypothesis: effect of employment status on alcohol consumption. Random-effects multinomial logistic regression.**

|  | Imputed Model (RE) (n=176,760) | | | | |
| --- | --- | --- | --- | --- | --- |
|  | Abstinence | |  | Binge Drinking | |
| (ref. Moderate drinking) | β | 95% CI |  | β | 95% CI |
| Employment status (employed) |  |  |  |  |  |
| Short unemployment | -0.05 | -0.15 to 0.05 |  | 0.06 | -0.04 to 0.17 |
| Long Unemployment | 0.38** | 0.29 to 0.46 |  | 0.34** | 0.24 to 0.44 |
| Occupational Disability | 0.59** | 0.51 to 0.66 |  | -0.04 | -0.15 to 0.07 |
| Gender (Men) |  |  |  |  |  |
| Women | 1.02** | 0.98 to 1.05 |  | -0.68** | -0.71 to -0.64 |
| Age | -0.02** | -0.02 to -0.02 |  | -0.05** | -0.05 to -0.05 |
| Health | -0.23** | -0.25 to -0.21 |  | -0.11** | -0.13 to 0.09 |
| Partner (Partnered/Married) |  |  |  |  |  |
| Single/Divorced/Widow | 0.34** | 0.29 to 0.39 |  | 0.57** | 0.52 to 0.61 |
| Years of Education | -0.12** | -0.13 to -0.12 |  | -0.17** | -0.18 to -0.16 |

**Table A4. Imputed model. Selection hypothesis: effect of alcohol consumption on employment status. Random-effects multinomial logistic regression.**

|  | Imputed Model (RE) (n=176,760) | | | | | | | |
| --- | --- | --- | --- | --- | --- | --- | --- | --- |
|  | Short Unemployment | |  | Long Unemployment | |  | Occup. Disability | |
| (ref. Employed) | β | 95% CI |  | β | 95% CI |  | β | 95% CI |
| Alcohol (moderate) |  |  |  |  |  |  |  |  |
| Abstinence | -0.04 | -0.14 to 0.06 |  | 0.37** | 0.29 to 0.45 |  | 0.51** | 0.43 to 0.59 |
| Binge Drinking | 0.09 | -0.01 to 0.19 |  | 0.37** | 0.27 to 0.47 |  | -0.03 | -0.14 to 0.08 |
| Gender (Men) |  |  |  |  |  |  |  |  |
| Women | -0.07 | -0.14 to 0.00 |  | 0.03 | -0.04 to 0.11 |  | -0.05 | -0.13 to 0.03 |
| Age | -0.03** | -0.03 to -0.02 |  | 0.04** | 0.04 to 0.05 |  | 0.05** | 0.05 to 0.06 |
| Health | -0.21** | -0.26 to -0.16 |  | -0.46** | -0.51 to -0.40 |  | -1.59** | -1.65 to -1.54 |
| Partner (Partnered/Married) |  |  |  |  |  |  |  |  |
| Single/Divorced/Widow | 0.75** | 0.67 to 0.85 |  | 1.32** | 1.23 to 1.39 |  | 0.85** | 0.77 to 0.94 |
| Years of Education | -0.10** | -0.11 to -0.08 |  | -0.13** | -0.15 to -0.12 |  | -0.15** | -0.17 to -0.13 |

**Table A5. Random- and Fixed-effects regression models with additional “Heavy Drinking” category in the outcome.**

|  | Model 1 (RE) (n=136,614) | | | | | | | | |  | | Model 2 (FE) (n=50,405) | | | | | | | | | | | | | | | | | | | |  |
| --- | --- | --- | --- | --- | --- | --- | --- | --- | --- | --- | --- | --- | --- | --- | --- | --- | --- | --- | --- | --- | --- | --- | --- | --- | --- | --- | --- | --- | --- | --- | --- | --- |
|  | Abstinence | |  | Heavy Drinking | |  | Binge Drinking | |  | | Abstinence | | | | | |  | Heavy Drinking | | | | | | |  | | Binge Drinking | | | | | |
| (ref. Moderate drinking) | β | 95% CI |  | β | 95% CI |  | β | 95% CI |  | | β | | 95% CI | | | |  | β | | 95% CI | | | | |  | | β | | 95% CI | | | |
| Employment status (employed) |  |  |  |  |  |  |  |  |  | |  | |  |  |  |  | | | | |  |  | | | |  | | | |  |  |  |
| Short unemployment | -0.10 | -0.22to0.02 |  | -0.08 | -0.25to0.08 |  | 0.03 | -0.11to0.17 |  | | -0.18 | | -0.41to0.05 | | | |  | -0.06 | | -0.34to0.23 | | | | |  | | -0.03 | | -0.28to0.22 | | | |
| Long Unemployment | 0.37** | 0.26to0.47 |  | 0.06 | -0.08to0.19 |  | 0.35** | 0.22to0.48 |  | | 0.24* | | 0.04to0.45 | | | |  | 0.09 | | -0.17to0.35 | | | | |  | | 0.40** | | 0.14to0.66 | | | |
| Occupational Disability | 0.61** | 0.52to0.70 |  | -0.13 | -0.26to0.01 |  | -0.04 | -0.17to0.09 |  | | 0.56** | | 0.31to0.81 | | | |  | -0.22 | | -0.58to0.14 | | | | |  | | -0.02 | | -0.36to0.32 | | | |
| Gender (Men) |  |  |  |  |  |  |  |  |  | |  | |  | | | |  |  | |  | | | | |  | |  | |  | | | |
| Women | 0.88** | 0.84to0.93 |  | -0.78** | -0.83to-0.73 |  | -0.81** | -0.86to-0.76 |  | |  | |  | | | |  |  | |  | | | | |  | |  | |  | | | |
| Age | -0.02** | -0.02to-0.02 |  | 0.03** | 0.03to0.03 |  | -0.04** | -0.05to-0.04 |  | | -0.06** | | -0.07to-0.06 | | | |  | -0.01** | | -0.02to-0.01 | | | | |  | | -0.05** | | -0.06to-0.05 | | | |
| Health | -0.24** | -0.27to-0.22 |  | -0.02 | -0.04to0.01 |  | -0.10** | -0.13to0.08 |  | | -0.07* | | -0.12to-0.01 | | | |  | 0.00 | | -0.06to0.06 | | | | |  | | 0.05 | | -0.01to0.11 | | | |
| Partner (Partnered/Married) |  |  |  |  |  |  |  |  |  | |  | |  | | | |  |  | |  | | | | |  | |  | |  | | | |
| Single/Divorced/Widow | 0.38** | 0.33to0.44 |  | 0.10** | 0.02to0.17 |  | 0.57** | 0.50to0.63 |  | | -0.15* | | -0.29to-0.01 | | | |  | 0.18* | | 0.00to0.35 | | | | |  | | 0.70** | | 0.56to0.85 | | | |
| Years of Education | -0.12** | -0.13to-0.11 |  | 0.02** | 0.01to0.03 |  | -0.17** | -0.17to-0.15 |  | |  | |  | | |  |  | |  | | | |  |  | | | |  | | |  |  |

**Table A6. Random- and Fixed-effects regression models with additional “Heavy Drinking” category in the independent variable.**

|  | Model 1 (RE) (n=136,614) | | | | | | | |  | Model 2 (FE) (n=12,879) | | | | | | | |
| --- | --- | --- | --- | --- | --- | --- | --- | --- | --- | --- | --- | --- | --- | --- | --- | --- | --- |
|  | Short Unemployment | |  | Long Unemployment | |  | Occup. Disability | |  | Short Unemployment | |  | Long Unemployment | |  | Occup. Disability | |
| (ref. Employed) | β | 95% CI |  | β | 95% CI |  | β | 95% CI |  | β | 95% CI |  | β | 95% CI |  | β | 95% CI |
| Alcohol (moderate) |  |  |  |  |  |  |  |  |  |  |  |  |  |  |  |  |  |
| Abstinence | -0.10 | -0.22to0.03 |  | 0.37** | 0.27to0.48 |  | 0.53** | 0.43to0.62 |  | -0.21 | -0.43to0.02 |  | 0.18 | -0.04to0.39 |  | 0.02 | -0.29to0.34 |
| Heavy Drinking | -0.08 | -0.24to0.08 |  | 0.10 | -0.04to0.23 |  | -0.08 | -0.22to0.05 |  | -0.03 | -0.32to0.25 |  | 0.05 | -0.23to0.32 |  | -0.26 | -0.72to0.20 |
| Binge Drinking | 0.04 | -0.10to0.18 |  | 0.37** | 0.24to0.50 |  | -0.02 | -0.15to0.11 |  | -0.03 | -0.28to0.21 |  | 0.39** | 0.12to0.65 |  | 0.12 | -0.31to0.54 |
| Gender (Men) |  |  |  |  |  |  |  |  |  |  |  |  |  |  |  |  |  |
| Women | 0.05 | -0.04to0.14 |  | 0.08 | -0.01to0.17 |  | -0.08 | -0.17to0.01 |  |  |  |  |  |  |  |  |  |
| Age | -0.01** | -0.02to-0.01 |  | 0.06** | 0.05to0.06 |  | 0.05** | 0.05to0.06 |  | 0.00 | -0.01to0.01 |  | 0.09** | 0.08to0.10 |  | 0.18** | 0.16to0.20 |
| Health | -0.18** | -0.24to-0.11 |  | -0.37** | -0.43to-0.31 |  | -1.59** | -1.65to-1.52 |  | 0.05 | -0.06to0.16 |  | -0.01 | -0.11to0.10 |  | -0.44** | -0.61to-0.28 |
| Partner (Partnered/Married) |  |  |  |  |  |  |  |  |  |  |  |  |  |  |  |  |  |
| Single/Divorced/Widow | 0.69** | 0.57to0.80 |  | 1.21** | 1.12to1.31 |  | 0.85** | 0.74to0.95 |  | 0.18 | -0.08to0.44 |  | 0.17 | -0.10to0.44 |  | 0.16 | -0.31to0.63 |
| Years of Education | -0.09** | -0.11to-0.07 |  | -0.12** | -0.14to-0.10 |  | -0.15** | -0.17to-0.13 |  |  |  |  |  |  |  |  |  |

**Table A7. Characteristics of the population included in Random- and Fixed*-*effects models*.***

|  | Alcohol consumption as outcome | | |  | Employment status as outcome | | |
| --- | --- | --- | --- | --- | --- | --- | --- |
|  | **Model 1 RE** (n=136,614) |  | **Model 2 FE** (n=38,922) |  | **Model 3 RE** (n=136,614) |  | **Model 4 FE** (n=12,879) |
| Gender (Women) | 54.82 |  | 59.20 |  | 54.82 |  | 57.15 |
| Age (mean; SD) | 48.91 (10.03) |  | 47.55 (10.44) |  | 48.91 (10.03) |  | 50.63 (10.47) |
| Years of Education (mean; SD) | 12.49 (2.28) |  | 12.23 (2.24) |  | 12.49 (2.28) |  | 11.90 (2.38) |
| Partner Status (Partnered) | 87.91 |  | 85.31 |  | 87.91 |  | 80.05 |
| Health (Very good/Excellent) | 38.33 |  | 35.01 |  | 38.33 |  | 27.09 |
| Abstinence | 17.74 |  | 27.03 |  | 17.74 |  | 21.78 |
| Binge Drinking | 10.64 |  | 21.63 |  | 10.64 |  | 11.41 |
| Long Unemployment | 2.37 |  | 2.60 |  | 2.37 |  | 20.77 |
| Occupational Disability | 3.64 |  | 4.20 |  | 3.64 |  | 12.22 |

**Table A8. Comparative models comparing binge drinkers with moderate drinkers (with no specific assessment for abstainers).**

|  | Model 1 (RE) (n=136,614) | | | | | | | |  | Model 2 (FE) (n=12,879) | | | | | | | |
| --- | --- | --- | --- | --- | --- | --- | --- | --- | --- | --- | --- | --- | --- | --- | --- | --- | --- |
|  | Short Unemployment | |  | Long Unemployment | |  | Occup. Disability | |  | Short Unemp. | |  | Long Unemp. | |  | Occup. Disability | |
| (ref. Employed) | β | 95% CI |  | β | 95% CI |  | β | 95% CI |  | β | 95% CI |  | β | 95% CI |  | β | 95% CI |
| Alcohol (moderate) |  |  |  |  |  |  |  |  |  |  |  |  |  |  |  |  |  |
| Binge Drinking | 0.07 | -0.06to0.21 |  | 0.27** | 0.15-0.39 |  | -0.16* | -0.29to0.03 |  | 0.00 | -0.24to0.23 |  | 0.36** | 0.10to0.62 |  | 0.17 | -0.25to0.58 |
| Gender (Men) |  |  |  |  |  |  |  |  |  |  |  |  |  |  |  |  |  |
| Women | 0.05 | -0.04to0.15 |  | 0.11* | 0.03to0.20 |  | 0.00 | -0.09to0.09 |  |  |  |  |  |  |  |  |  |
| Age | -0.01** | -0.02- -0.01 |  | 0.05** | 0.05to0.06 |  | 0.05** | 0.05to0.06 |  | 0.00 | -0.01to0.01 |  | 0.09** | 0.08to0.10 |  | 0.18** | 0.16to0.20 |
| Health | -0.17** | -0.24to-0.11 |  | -0.38** | -0.44to-0.32 |  | -1.63** | -1.69to-1.56 |  | 0.05 | -0.06to0.16 |  | -0.01 | -0.12to0.10 |  | -0.44** | -0.61to-0.28 |
| Partner (Partnered/Married) |  |  |  |  |  |  |  |  |  |  |  |  |  |  |  |  |  |
| Single/Divorced/Widow | 0.68** | 0.57to0.80 |  | 1.24** | 1.14to1.33 |  | 0.89** | 0.79to0.99 |  | 0.19 | -0.07to0.45 |  | 0.17 | -0.10to0.44 |  | 0.15 | -0.31to0.62 |
| Years of Education | -0.09** | -0.11to-0.07 |  | -0.12** | -0.14to-0.11 |  | -0.16** | -0.18to-0.14 |  |  |  |  |  |  |  |  |  |

**Figure A1 – Reciprocal causality between long unemployment spells, binge drinking, and abstinence (allowing the coefficients to vary across waves).**


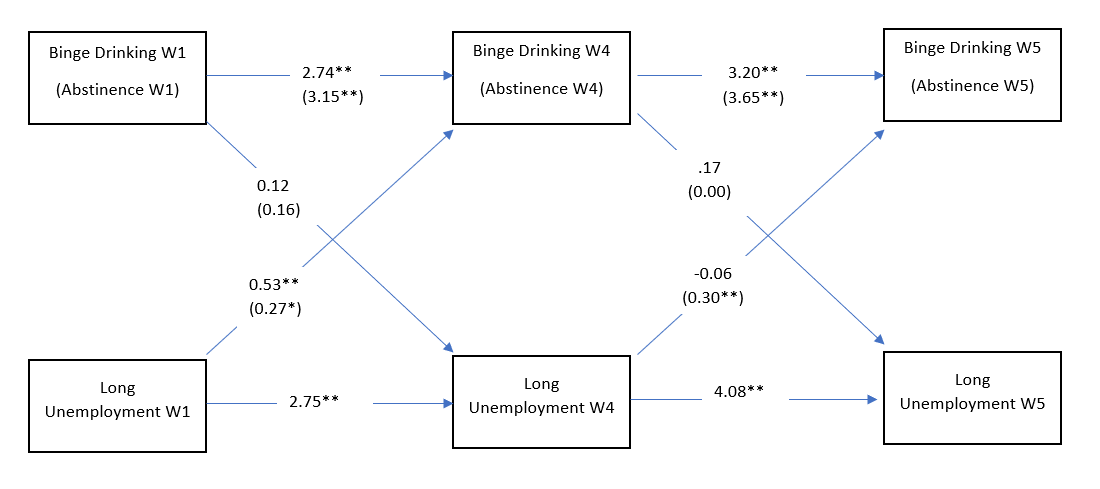


**Figure A2 – Reciprocal causality between occupational disability, binge drinking, and abstinence (allowing the coefficients to vary across waves).**


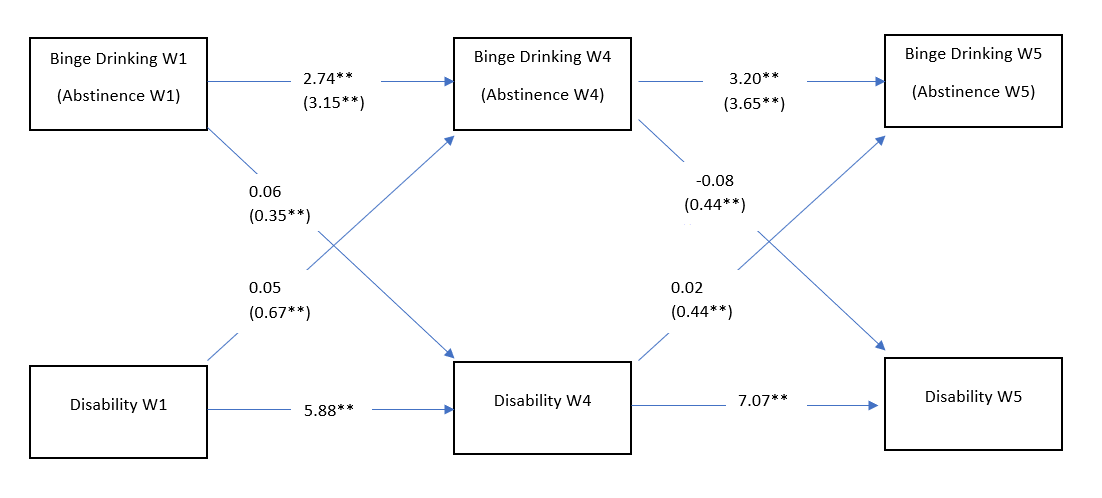


**Appendix #2. Fixed-effects cross-lagged GSEM models.**

In this part of the Appendix, the fixed-effects cross-lagged GSEM model as discussed by Allison at al. (2017) is outlined.

As shown in Figure A3, in these models the independent variables precede the outcome in time (e.g. unemployment at wave 1 affects binge drinking at wave 4, and vice versa). Thus, alcohol consumption and employment status both as outcome, as well as main independent variable.


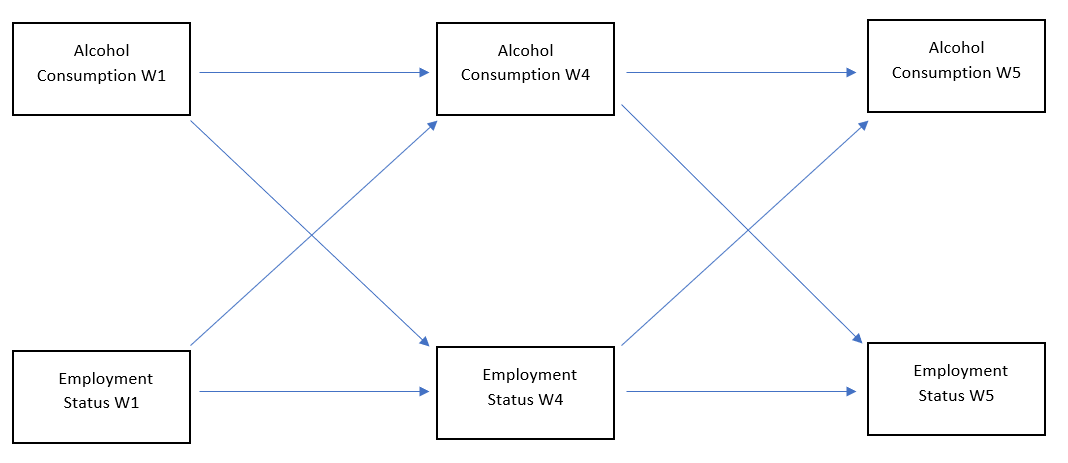


**Figure A3. Cross-lagged panel model.**

Moreover, we estimated these models accounting for fixed-effects, according to the following form (Allison, et. al., 2017):

*Y_it_=µ_t_+* *β_1_x_i,t-1_ + β_2_y_i,t-1_ +* δ_1_*w*_it_ + γ_1_*z*_i_ *+*α_i_+ε_it_

*x_it_=*τ*_t_+* *β_3_x_i,t-1_ + β_4_y_i,t-1_ +* δ_2_*w*_it_ + γ_2_*z*_i_ *+*η_i_+ε_it_

where (*x_i,t-1_*) and (*y_i,t-1_*) are the cross-lagged effects of *x* and *y*; *w*_it_ are the time-varying covariates, and *z_i_* are the observed time-constant covariates -which drop out of the model, hence the absence of a *β_i_z_i_* term-, and α_i_ and η*_i_* represent unobserved time-constant heterogeneity. Please note that *z_i,_* α_i,_ and η*_i_* vary between individuals, but do not change over time for one individual (hence they have no *t* subscript) (Bollen & Brand, 2008).

Last, in our fixed-effects models, both time-constant unobserved components (α_i,_ and η*_i_*) are fixed to 1. Moreover, two additional equations were added in order to allow them to correlate with the time-varying covariates (the code used is available in the Appendix):

α_i=_ *β_1_w_i,t-1_ + β_2_w_i,t-1_* + ε_it_

η*_i=_ β_1_w_i,t-1_ + β_2_w_i,t-1_* + ε_it_

In the results in the main body of our article, the model was constrained so that the effect between t and t+1 would be the same between baseline and wave 4, and between wave 4 and wave 5, i.e. it is assumed that the coefficients of the covariates remain equal across all waves of data (Bollen & Brand, 2008). An alternative model, in which the coefficients are allowed to vary between waves, was run as sensitivity analysis and is shown in the appendix #1 (see Figures A1 and A2).
