# Supplementary material for: Disentangling the association between alcohol consumption and employment status: causation, selection or confounding?
Source: Eur J Public Health. 2022 Oct 10;32(6):926–32. doi: 10.1093/eurpub/ckac141 (PMC9713390; doi:10.1093/eurpub/ckac141)
Supplement: ckac141_Supplementary_Data [file ckac141_supplementary_data.zip › ejph-2021-11-om-1082-File006.docx]

**Stata syntax**

1. **Random and Fixed effects regression models**

.xtset PSEUDOIDEXT Wave

*RANDOM EFFECTS*

***Causation*

.mlogit BDcat i.Work5 ib1.Partner Age_c Health Gender Years_Education, vce(cluster PSEUDOIDEXT)

*** Selection*

.mlogit Work5 ib1.BDcat ib1.Partner Age_c Health Gender Years_Education, vce(cluster PSEUDOIDEXT)

FIXED EFFECTS

*** Causation*

.femlogit BDcat ShortUnemp LongUnemp2 Unfit Age_c Partner2 Health

*** Selection*

.femlogit Work5 Abstainer BD Age_c Partner2 Health

*Comparison between the samples in the RE and FE models:*

.mlogit Work5 ib1.BDcat ib1.Partner Age_c Health Gender Years_Education, vce(cluster PSEUDOIDEXT)

.predict p1 if e(sample)==1

.gen SelectRE=1 if p1!=.

.replace SelectRE=0 if SelectRE==.

.femlogit Work5 Abstainer BD Age_c Partner2 Health

.predict p2 if e(sample)==1

.gen SelectFE=1 if p2!=.

.replace SelectFE=0 if SelectFE==.

.mlogit BDcat i.Work5 ib1.Partner Age_c Health Gender Years_Education, vce(cluster PSEUDOIDEXT)

.predict p3 if e(sample)==1

.gen CausatRE=1 if p3!=.

.replace CausatRE=0 if CausatRE==.

.femlogit BDcat ShortUnemp LongUnemp2 Unfit Age_c Partner2 Health

.predict p4 if e(sample)==1

.gen CausatFE=1 if p4!=.

.replace CausatFE=0 if CausatFE==.

1. **Generalized Structural Equation Models with Fixed Effects**

**Coefficients constrained be the same across different waves.*

.gsem (shortunempw4 <- shortunempw1@a longunemp2w1@b unfitw1@c bdw1@d abstainerw1@e healthw1@f agew1@g partner2w1@h Alpha@1, logit) (longunemp2w4 <- shortunempw1@i longunemp2w1@j unfitw1@k bdw1@l abstainerw1@m healthw1@n agew1@o partner2w1@p Alpha@1, logit) (unfitw4 <- shortunempw1@q longunemp2w1@r unfitw1@s bdw1@t abstainerw1@u healthw1@v agew1@x partner2w1@y Alpha@1, logit) (shortunempw5 <- shortunempw4@a longunemp2w4@b unfitw4@c bdw4@d abstainerw4@e healthw4@f agew4@g partner2w4@h Alpha@1, logit) (longunemp2w5 <- shortunempw4@i longunemp2w4@j unfitw4@k bdw4@l abstainerw4@m healthw4@n agew4@o partner2w4@p Alpha@1, logit) (unfitw5 <- shortunempw4@q longunemp2w4@r unfitw4@s bdw4@t abstainerw4@u healthw4@v agew4@x partner2w4@y Alpha@1, logit) (Alpha <- healthw1 healthw4 age* partner2*) (bdw4 <- bdw1@aa abstainerw1@bb shortunempw1@cc longunemp2w1@dd unfitw1@ee healthw1@ff agew1@gg partner2w1@hh Alpha@1, logit) (abstainerw4 <- bdw1@ii abstainerw1@jj shortunempw1@kk longunemp2w1@ll unfitw1@mm healthw1@nn agew1@oo partner2w1@pp Alpha@1, logit) (bdw5 <- bdw4@aa abstainerw4@bb shortunempw4@cc longunemp2w4@dd unfitw4@ee healthw4@ff agew4@gg partner2w4@hh Alpha@1, logit) (abstainerw5 <- bdw4@ii abstainerw4@jj shortunempw4@kk longunemp2w4@ll unfitw4@mm healthw4@nn agew4@oo partner2w4@pp Alpha@1, logit)

.est store GSEM1

**Coefficients allowed to differ across waves*

.gsem (shortunempw4 <- shortunempw1@a longunemp2w1@b unfitw1@c bdw1@d abstainerw1@e healthw1@f agew1@g partner2w1@h Alpha@1, logit) (longunemp2w4 <- shortunempw1@i longunemp2w1@j unfitw1@k bdw1@l abstainerw1@m healthw1@n agew1@o partner2w1@p Alpha@1, logit) (unfitw4 <- shortunempw1@q longunemp2w1@r unfitw1@s bdw1@t abstainerw1@u healthw1@v agew1@x partner2w1@y Alpha@1, logit) (shortunempw5 <- shortunempw4@aaa longunemp2w4@bbb unfitw4@ccc bdw4@ddd abstainerw4@eee healthw4@fff agew4@ggg partner2w4@hhh Alpha@1, logit) (longunemp2w5 <- shortunempw4@iii longunemp2w4@jjj unfitw4@kkk bdw4@lll abstainerw4@mmm healthw4@nnn agew4@ooo partner2w4@ppp Alpha@1, logit) (unfitw5 <- shortunempw4@qqq longunemp2w4@rrr unfitw4@sss bdw4@ttt abstainerw4@uuu healthw4@vvv agew4@xxx partner2w4@yyy Alpha@1, logit) (Alpha <- healthw1 healthw4 age* partner2*) (bdw4 <- bdw1@aa abstainerw1@bb shortunempw1@cc longunemp2w1@dd unfitw1@ee healthw1@ff agew1@gg partner2w1@hh Alpha@1, logit) (abstainerw4 <- bdw1@ii abstainerw1@jj shortunempw1@kk longunemp2w1@ll unfitw1@mm healthw1@nn agew1@oo partner2w1@pp Alpha@1, logit) (bdw5 <- bdw4@aax abstainerw4@bbx shortunempw4@ccx longunemp2w4@ddx unfitw4@eex healthw4@ffx agew4@ggx partner2w4@hhx Alpha@1, logit) (abstainerw5 <- bdw4@iix abstainerw4@jjx shortunempw4@kkx longunemp2w4@llx unfitw4@mmx healthw4@nnx agew4@oox partner2w4@ppx Alpha@1, logit)

.est store GSEM2

**Compare coefficients*

.estimates table GSEM1 GSEM2, b(%12.2fc)eform star stfmt(%-9.3f)stats(N)

1. **Multiple Imputation (MICE)**

.drop if Wave==2 | Wave==3

.mi set mlong

.mi register impute BDcat Work5 Health Years_Education Partner Age_c

.mi impute chained (mlogit)BDcat (mlogit)Work5 (mlogit)Health (truncreg, ll(5) ul(16))Years_Education (logit)Partner (truncreg, ll(22) ul(75))Age_c = Gender, add(40) augment force

**Estimates based on observed values in the outcome*

**Random effects*

.preserve

.contract PSEUDOIDEXT Wave BDcat Work5 Age_c Gender Partner Years_Education Health _mi_miss _mi_m _mi_id

.sort PSEUDOIDEXT Wave _mi_m

.bysort PSEUDOIDEXT Wave (_mi_m) : replace _mi_miss = _mi_miss[_n-1] if missing(_mi_miss)

.replace BDcat=. if _mi_miss==1

.mi estimate: mlogit BDcat i.Work5 i.Gender c.Age_c ib1.Partner Years_Education Health, base(1) vce(cluster PSEUDOIDEXT)

.restore

.preserve

.contract PSEUDOIDEXT Wave BDcat Work5 Age_c Gender Partner Years_Education Health _mi_miss _mi_m _mi_id

.sort PSEUDOIDEXT Wave _mi_m

.bysort PSEUDOIDEXT Wave (_mi_m) : replace _mi_miss = _mi_miss[_n-1] if missing(_mi_miss)

.replace Work5=. if _mi_miss==1

.mi estimate: mlogit BDcat i.Work5 i.Gender c.Age_c ib1.Partner Years_Education Health, base(1) vce(cluster PSEUDOIDEXT)

.restore

*Fixed-effects

.mi passive: gen BDimp=1 if BDcat==2

.mi passive: replace BDimp=0 if BDimp==.

.mi passive: gen Abstimp=1 if BDcat==0

.mi passive: replace Abstimp=0 if Abstimp==.

.mi xtset PSEUDOIDEXT Wave

.mi estimate: xtlogit BDimp i.Work5 Abstimp c.Age_c ib1.Partner Health, fe
